# Comparison of Different Home/Commercial Washing Strategies for Ten Typical Pesticide Residue Removal Effects in Kumquat, Spinach and Cucumber

**DOI:** 10.3390/ijerph16030472

**Published:** 2019-02-06

**Authors:** Yangliu Wu, Quanshun An, Dong Li, Jun Wu, Canping Pan

**Affiliations:** Department of Applied Chemistry, College of Science, China Agricultural University, Beijing 100193, China; wuyangliu@cau.edu.cn (Y.W.); anquanshun1@163.com (Q.A.); lidong1105@cau.edu.cn (D.L.); wujun@cau.edu.cn (J.W.)

**Keywords:** pesticide residues, washing process, alkaline electrolyzed water, active oxygen, micron calcium solution

## Abstract

Home processing can reduce pesticide residues in agricultural products, and the common forms of treatment include washing, peeling, blanching, and cooking. In this study, the removal effects of tap water, micron calcium solution, alkaline electrolyzed water (AlEW), ozone water, active oxygen, and sodium bicarbonate on 10 typical pesticide residues in kumquat, cucumber, and spinach were investigated. The residue magnitudes were determined by chromatography–tandem mass spectrometry (GC-MS/MS, LC-MS/MS), combined with the QuEChERS pretreatment method. The model tests showed that the results of soaking and greenhouse were close. The removal effects of pesticide residues in kumquat and cucumber washing by alkaline electrolyzed water with a high pH value, micron calcium, and active oxygen solution were better than other washing solutions. The sodium bicarbonate solution, ozone water, and active oxygen solution were more effective in reducing pesticide residues in spinach than others. Active oxygen solution showed a better removal efficiency for the 10 pesticides than other treatments because of its alkalinity and oxidizability. Among the ten pesticides, pyrethroid pesticides had a higher removal rate. Additionally, chlorpyrifos were the most difficult to remove. For the majority of pesticides, the pesticide residue magnitudes showed a gradual reduction when increasing the washing time. The results indicated that alkaline solutions were effective for the reduction of pesticide residues when the washing time was longer than 15 min.

## 1. Introduction

Pesticides are used to control plant diseases, insect pests, and weeds and regulate plant growth to ensure the quality and quantity of the produce. Pesticides are not made up of one component, but consist of several mixtures and adjuvants. Excessive pesticide residues can do great harm to customers, with effects such as neurotoxicity, carcinogenicity, reproduction abnormality, and cell dysplasia. Nowadays, pesticide remnant is still a major problem affecting the quality and security of fruits and vegetables.

Food processing, such as washing, peeling, blanching, and cooking, plays a common role in the reduction of residues. Washing is the most common and direct form of food processing, is usually the first step before consumption, and is used for removing pesticide residues in fruits and vegetables [1,2]. After harvest, some kinds of produce, such as fresh fruits and vegetables, are often washed with tap water to remove dirty marks on the surface, which are then consumed directly. However, tap water has a limited effect on the removal of pesticide residues, because many pesticides are hydrophobic [3]. Therefore, many detergent solutions are used to degrade pesticides in vegetables and fruits, including sodium chloride solution, acetic acid, sodium carbonate, and sodium bicarbonate. Y. Liang [4] studied the removal of five organophosphorus pesticides in raw cucumber with home preparation, and the research results show that washing by tap water for 20 min only caused a pesticides reduction of 26.7–62.9%. Sodium carbonate and sodium bicarbonate solution caused a pesticides reduction of 66.7–98.9%. Storage in low temperature caused a pesticides reduction of 60.9–90.2% and ultrasonic cleaning for 20 min lowered pesticides by 49.8–84.4% in raw cucumber. Apart from the common detergent solutions, ozone is also used for the removal of the pesticides residue in fruits and vegetables such as carrot, Chinese white cabbage and greenstem bok choy [5], and orange [6], without modifying its physicochemical property and organoleptic characteristics [7]. The highest removal percentages of tetradifon and chlorpyrifos ethyl in lemon and grapefruit matrices that have been achieved with ozonation are 98.6% and 94.2%, respectively. Ozone can also degrade some pesticides in natural waters [8,9]. Meanwhile, electrolytic water is also widely investigated as a disinfectant and detergent in the food industry. There are two types of electrolytic water. Electrolyzed oxidizing (EO) water is extensively used with a low pH and high oxidation–reduction potential. Electrolyzed reduced (ER) water has limited application due to its characteristic of a high pH and low ORP [10,11]. However, researchers found that the ER water could be used as a cleaning solution to reduce pesticide residues in fruits and vegetables; for example, cabbage, leek [2], beans, grapes [3], and cowpea [11]. The removal of six pesticides in cowpea washing by AlEW solution (pH = 12.2) for 45 min was 48–85%. Based on previous studies, alkaline electrolyzed water with two different pH values was selected as a cleaning agent.

**Figure 1:**
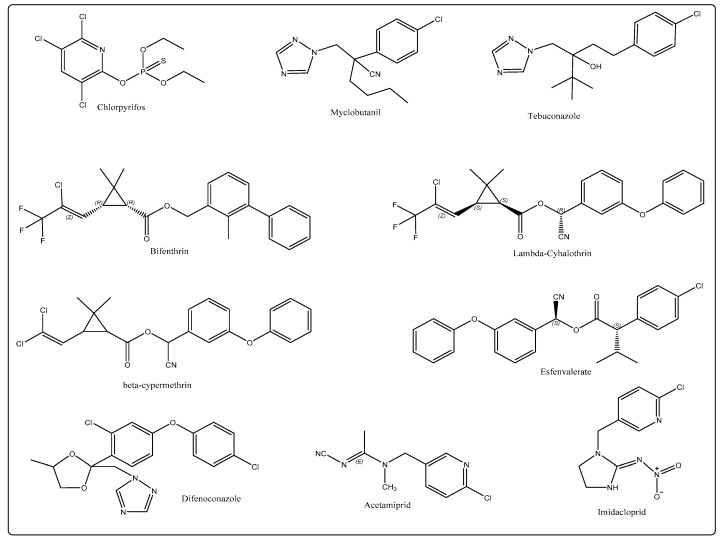
The chemical structures of the studied pesticides.

In this study, the objective was to evaluate the effectiveness of detergent solution in removing the pesticides organophosphates, triazoles, pyrethroids, and neonicotinoids from fresh kumquat, spinach, and cucumber, which are widely used to control pests and diseases in fruits and vegetables. Kumquat and cucumber can be washed and directly eaten without peeling and cooking. Spinach is one of the most common vegetables in daily life and is very nutritional. Therefore, it is of great significance to study the removal of pesticide residues in kumquat, cucumber, and spinach with washing treatments. The main physic-chemical properties and chemical structures of the studied pesticides are presented in Table 1 and Figure 1, respectively. The common home preparation, tap water, and sodium bicarbonate solution [12], were used for comparison with alkaline electrolyzed water and ozone water, which have often been reported in the literature. At the same time, the micron calcium and active oxygen of the pesticide removal products on the market were also compared. Different treatment methods were used to clean the matrix for 5, 15, 20, and 30 min, combined with the QuEChERS pretreatment method [13], as well as chromatography-mass spectrometry technology, in order to find the best cleaning method and the best cleaning time. At the same time, the impact of different treatment methods and pesticides on different substrates was explored.

## 2. Materials and Methods

### 2.1. Standards, Reagents, and Materials

The purities of the ten pesticide standards were from 97% to 99%, which were obtained from the Institute of Control of Agrochemicals, Ministry of Agriculture People’s Republic of China. Standard stock solutions (500 mg/L) for mixture of the ten pesticides were prepared in acetonitrile and stored at −20 °C. The work solution was prepared daily. Acetonitrile was of chromatography grade and obtained from Fisher Scientific (Fair Lawn, NJ, USA). Sodium chloride (NaCl) and anhydrous magnesium sulfate (MgSO_4_) were of analytical grade and purchased from Sinopharm Chemical Reagent Co., Ltd. (Beijing, China). Multi-walled carbon nanotubes (MWCNTs) with average external diameters of 10–20 nm and primary secondary amine (PSA) (40 µm) were acquired from Agilent Technology Co., Ltd. (Beijing, China). Micron calcium was provided by Bai Jia An Bioengineering Co., Ltd. (Liaoning, China). Active oxygen was provided by Guangzhou Zao Gu Biotechnology Co., Ltd. (Guangzhou, China). Alkaline electrolyzed water and ozonated water were prepared by the Specialized preparation machine.

Centrifugation was performed in an Anke TDL–40 B centrifuge equipped with a bucket rotor (8 × 100 mL) (Shanghai, China). An ATARGIN VX–III multitube vortexer was used in sample preparation (Beijing, China).

### 2.2. GC–MS/MS Analysis

The analysis of the pesticides was carried out with the Thermo Scientific TSQ 8000 EVO triple quadrupole mass spectrometer coupled with a Trace 1300 gas chromatograph and a TriPlus AI 1310 autosampler (Thermo Fisher Scientific, San Jose, CA, USA). An Agilent Technologies capillary column (30 m × 250 μm × 0.25 μm film thickness) was used for chromatographic separation. The column temperature was initially set at 40 °C and held for 0.4 min, and then increased to 180 °C at the rate of 30 °C/min, 280 °C at the rate of 10 °C/min, and finally 290 °C at the rate of 20 °C/min and held for 5 min. The temperature of the injector port was 250 °C and the injection volume was 1 μL. The total running time was 25 min. Helium gas was used as the carrier gas, with a constant flow of 1.0 mL/min, and Argon gas was chosen as the collision gas, with the pressure of 1.5 mTorr. The mass spectrometer was operated in electron ionization (EI) mode at 70 eV. The ion source and transfer line temperatures were set at 280 °C and 280 °C, respectively. Table 2 summarizes the condition of mass spectrum [14,15] and the typical retention time for each analyte.

### 2.3. LC–MS/MS Analysis

The column of the liquid chromatography was Athena C18-WP (2.1 mm × 50 mm × 3 μm, Agilent, Santa, Clara, CA, USA). The mobile phase was acetonitrile and 0.1% formic acid-water solution and the ratio was 4:6. The flow rate was 0.2 mL/min. The column was kept at 30 °C with the injection volume at 10 μL. A liquid chromatography-tandem mass spectrometer (Agilent 6410B, Agilent, -Santa Clara, CA, USA) coupled with a positive electrospray ionization (ESI+) source using multiple reaction monitoring mode (MRM) was used for analysis. The nitrogen was used as dry gas and atomization gas and the flow rate was 8.0 L/min. The gas temperature was 350 °C and the nebulizer pressure was 35 psi. A summary of the transitions monitored [16], the fragmentor voltage, and the collision energy parameters for acetamiprid and imidacloprid are given in Table 3.

### 2.4. Sample Preparation and Washing

Fresh vegetables of spinach, cucumber, and kumquat were purchased from a supermarket in Beijing, China. The kumquat and cucumber were steeped in 5 L mixed solution (50 mg/L), which was prepared with the ten pesticide formulations for 15 min. The spinach was immersed in 10 L mixed solution (10 mg/L) for 15 min. The contaminated kumquat, cucumber, and spinach were air-dried in a fume hood for 24 h at room temperature.

After that, 100 g of spinach, cucumber, and kumquat was randomly selected to detect the initial deposits. The polluted sample was washed by six washing methods (Tap water, AlEW solution (pH 12.35, pH 10.5), micron calcium water (10 g micron calcium and 500 mL tap water), 0.4 mg/kg ozone water, 2% active oxygen solution, and 2% NaHCO_3_) for 5, 15, 20, and 30 min, respectively. The washed samples were rinsed by tap water for 30 s. Following this, the treated samples were air-dried at room temperature and then analyzed.

### 2.5. Extraction and Purification of Pesticides

The cucumber, spinach, and kumquat were homogenized and processed by the blender, respectively. An amount of (10 ± 0.05 g) homogenized samples was weighed into a 50 mL centrifuge tube, and 10 mL acetonitrile was added. The resulting solution was shaken by the vortex for 5 min. After that, 1 g of sodium chloride and 4 g of anhydrous Magnesium Sulfate were added. The tube was cooled to room temperature and was then shaken for 5 min before centrifugation for 5 min at 3800 rpm. An aliquot of 1 mL supernatant of kumquat was transferred to a 2 mL centrifuge tube containing 5 mg MWCNTs and 30 mg PSA mixed with 150 mg anhydrous MgSO_4_ (The sorbent of spinach was 7.5 mg MWCNTs mixed with 150 mg anhydrous MgSO_4_ and the cucumber was 5 mg MWCNTs mixed with 150 mg anhydrous MgSO_4_). Then, the 2 mL tube was shaken for 1 min and centrifuged for 2 min at 4000 rpm. Finally, the supernatant was filtered through a 0.22 μm membrane into an autosampler vial for analysis.

### 2.6. Methods Validation

The validation was performed on each matrix, and the method was validated through linearity, the matrix effect, trueness and precision, limit of detection (LOD), and limit of quantification (LOQ). Linearity of the method was studied at five concentrations in the range of 10–1000 μg/kg for 10 pesticides by matrix-matched calibration solutions. Good linearity was found for the pesticides with coefficients of determination (R^2^) better than 0.990 [14]. LOQs for 10 pesticides were the lowest spike level of the method’s validation and the LOQs were regarded as LODs in this respect [17]. All data are shown in Table 4.

The accuracy was evaluated by recovery and the precision was evaluated by the relative standard deviation (RSD). This study was performed at three concentration levels (10, 100, and 500 μg/kg) by spiking standard pesticides for a blank sample. The results are shown in Table 5. The average recoveries of the10 pesticides were in the range of 78 to 118% and the RSDs were <10%.

## 3. Results and Discussion

### 3.1. Establishment of Soaking Model

**Figure 2:**
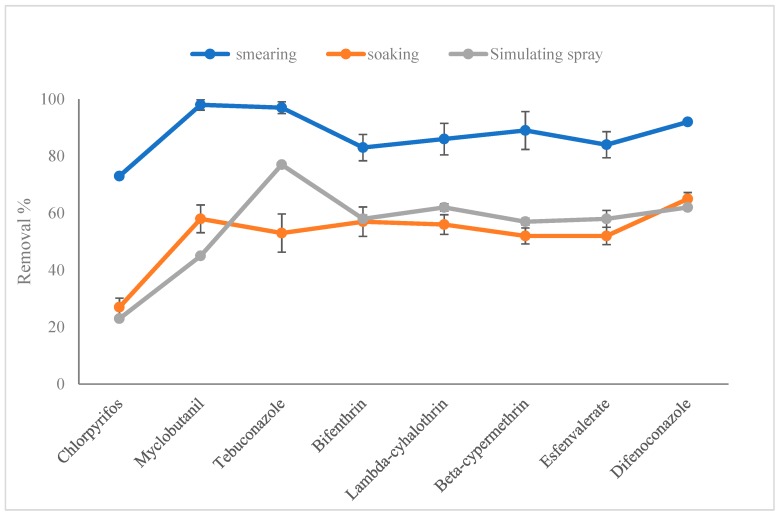
Removal efficiency of eight pesticides in kumquat treated with smearing, soaking, or simulating the field application by washing with 5 g micron calcium for 15min (*n* = 3).

Taking kumquat as the research object, three models of smearing, soaking, and simulating the field application resulted in eight pesticides being attached to the kumquat. The treatment of the washing matrix with prepared solution (5 g micron calcium and 500 mL tap water) for 15 min was conducted to compare the removal efficiency of eight pesticides, as can be seen in Figure 2. Three models caused a 73–99%, 27–65%, and 23–77% loss of the eight pesticides, respectively. The results of soaking and simulating the field application were close to each other. Thus, the model of soaking was chosen to carry out the next experiment.

Smearing: The pesticide was divided into two groups. The mixture of pesticide formulations (200 mg/L) was mixed with acetone. A syringe was used to remove a mixed solution of 1 mL from the surface of the kumquat. A sample of kumquat (of about 100 g) was determined, and was placed for an hour at room temperature.

Soaking: The kumquat was steeped in 2 L mixed solution (50 mg/L) for 15 min, which was prepared with the eight pesticide formulations. A sample of kumquat (of about 100 g) was determined, and was placed at room temperature for 24 h.

Simulating the field application: The eight pesticides were divided into two groups. According to the highest recommended dosage, two mixed solutions (500 mL) were configured, with four pesticide formulations for each. Then, the mixed solution was sprayed on a group of trees. A group consisted of two kumquat trees. After three days, approximately 200 g kumquat was picked from each group (100 g was cleaned, 100 g was control), and no spray was used in the blank control.

### 3.2. Effect of Washing Treatments for Pesticide Removal in Kumquat

Tap water and alkaline solution are relatively common washing solutions in our daily lives. The alkaline electrolyzed water (AlEW) is of high pH value and low oxidation reduction potentials, which is gradually being valued [11,18,19,20,21]. Ozone and active oxygen have a strong oxidation capability, which can destroy the unsaturated bonds and oxidize functional groups to decompose most organic compounds, and they do not produce secondary pollutants [22,23]. In this study, the effects of washing by tap water, 2% sodium bicarbonate solution, alkaline electrolyzed water, Micron calcium solution, ozone water (0.4 mg/kg), and 2% active oxygen solution for 5, 15, 20, and 30 min were investigated, and the washing results for kumquat are shown in Table 6. Washing with tap water, as well as detergent solutions, had an effect in reducing pesticide residue in kumquat. The removal of 10 pesticides in kumquat is 20–40% by tap water washing, and the effects of tap water for acetamiprid, imidacloprid, myclobutanil, and tebuconazole were superior to others, which is related to the O/W partition coefficient of pesticides.

Among these washing processing methods, 2% sodium bicarbonate solution and ozone water caused 20–40% more loss of the 10 pesticides than tap water. The removal effect of the AlEW, whose pH value was 12.35, was better than the one whose pH was 10.50. Micron calcium solution and 2% active oxygen solution were the most effective ways for the elimination of pesticide residues in kumquat. The greatest loss of chlorpyrifos, beta-cypermethrin, and esfenvalerate was 51%, 71%, and 54%, respectively, which was caused by micron calcium solution. The 2% active oxygen solution caused the lowest residual amounts of myclobutanil, tebuconazole, bifenthrin, lambda-cyhalothrin, difenoconazole, acetamiprid, and imidacloprid, which were reduced by 79%, 79%, 65%, 74%, 64%, 59%, and 67%, respectively. The residues of pyrethroid pesticides were the lowest and there was no significant difference on pesticide residue after 20 min washing treatment. Pesticide residues in fruits and vegetables showed a gradual reduction when increasing the treatment time, which is in agreement with Y. Liang [4] and Zhi-Yong Zhang [24].

### 3.3. Effect of Washing Treatments for Pesticide Removal in Cucumber

The results of the washing solution for removing pesticide residue in cucumber are presented in Table 7. Washing with tap water was a little effective in reducing pesticides in cucumber, and the removal rates of 10 pesticides were less than 35%. The removal effects of AlEW (pH 10.5) and ozone water were not obvious, unlike the pesticides in cucumber, which were effectively removed by washing with AlEW (pH 12.35), micron calcium, and active oxygen, and the removal rate of pyrethroid pesticides was obviously higher than others. Washing with 2% active oxygen solution for 20 min caused a 49%, 41%, 40%, 57%, 58%, 51%, 63%, 53%, 49%, and 50% loss in chlorpyrifos, myclobutanil, tebuconazole, bifenthrin, lambda-cyhalothrin, beta-cypermethrin, esfenvalerate, difenoconazole, acetamiprid, and imidacloprid, respectively. Washing with micron calcium solution for 20 min caused a greater loss of pesticides in cucumber, and the removal efficiency was 50%, 42%, 47%, 67%, 83%, 85% ,86%, 67%, 37%, and 35%, respectively.

### 3.4. Effect of Washing Treatments for Pesticide Removal in Spinach

The effect of washing treatments for pesticide removal in spinach are summarized in Table 8. It was difficult to remove pesticides from spinach by tap water, and the order of the removal effects of 10 pesticides in spinach by washing with detergent solution was as follows: ozone water and active oxygen solution > micron calcium solution >AlEW (pH 12.35) and sodium bicarbonate solution > AlEW (pH 10.50) > tap water. These washing methods are two to four times as effective as tap water. The residual amounts of chlorpyrifos, myclobutanil, tebuconazole, bifenthrin, lambda-cyhalothrin, beta-cypermethrin, esfenvalerate, difenoconazole, acetamiprid, and imidacloprid in spinach, which was washed with ozone water for 30 min, were reduced by 53%, 72%, 73%, 62%, 67%, 65%, 78%, 68%, 64%, and 63%, respectively. After being washed with active oxygen solution for 5 min, the removal efficiency of chlorpyrifos, myclobutanil, tebuconazole, bifenthrin, lambda-cyhalothrin, beta-cypermethrin, esfenvalerate, difenoconazole, acetamiprid, and imidacloprid in spinach was 52%, 63%, 65%, 55%, 70%, 71%, 81%, 62%, 50%, and 48%, respectively. According to the experimental result, the pesticides in spinach were easier to remove by oxidizing washing solution.

The optimal treatments of pesticides are shown in Table 9. Active oxygen, micron calcium, and ozone solution are the most effective treatments for kumquat, cucumber, and spinach, respectively. Tap water has a better removal effect on the 10 pesticides in cucumber, and the removal effect of the 10 pesticides in spinach is poor. The effect of 2% active oxygen solution treatment for pesticide removal in kumquat and spinach was superior to cucumber. Micron calcium solution (10 g micron calcium and 500 mL tap water) can effectively remove 10 pesticide residues in kumquat, cucumber, and spinach, and has a pH value of 12.93. The removal efficiency of pesticides from fruits and vegetables by 2% active oxygen solution is better than others because of its alkalinity (pH 10.88) and oxidizability. The pyrethroid pesticides had a higher removal rate as a result of their instability in alkaline solution. These results show that the removal rate of pesticides is associated with the pH of the washing solution, the pesticide properties, and the type of fruits and vegetables.

## 4. Conclusions

The removal effects of ten pesticide residues in kumquat, cucumber, and spinach when using different detergent solutions were investigated. After soaking, the deposition of pesticides in fruits and vegetables were different, which made the experimental data generate an inevitable error. However, the overall trend is obvious. Pesticide residues in fruits and vegetables showed a gradual reduction when increasing the treatment time for the majority of pesticides. It was obvious that the removal effect of washing for 15 min was vastly different from 5 min, and there was no significant difference in pesticide residue after 15 min washing treatment. Pesticides in cucumber were more easily removed by alkaline solutions, such as AlEW, micron calcium, and sodium bicarbonate solution, compared with oxidizing solutions. On the contrary, the pesticides in spinach were easily removed by oxidizing solutions. The removal efficiency of other washing solutions outperformed the tap water; tap water washing only caused a 10–40% loss of the 10 pesticides, and the AlEW, micron calcium, and active oxygen solution caused a 40–90% loss of the 10 pesticides. The data indicated that the lower Kow the pesticides had, the easier they were removed by washing with tap water, but it was inadequate when washing with other solutions. Pyrethroid pesticides adhering to plant superficies were removed more easily by washing, which is instable in the presence of alkaline solution and sunlight. The removal percentage of pesticides depended on the different washing solutions and the time of treatment, as well as the characteristics of pesticides, such as the lower octanol–water partition coefficient (Kow), mode of action, and the stability of hydrolysis and photolysis. These results clearly indicate that washing samples with detergent solution could effectively reduce pesticide residues in fruits and vegetables and ensure that humans have a healthy diet. Though the removal effects of different washing treatments have been studied in the existing literature, there has been no further study on the effects of detergents on the quality of fruit and vegetable and human health. The effect of cleaning agent residues will be studied in future work.

## Figures and Tables

**Table 1:** The main properties of the studied pesticides.

Pesticides	Category	LogP *	Stability	Water Solubility at 20 °C (mg/L)
Chlorpyrifos	Insecticide	4.7	Rate of hydrolysis increases with pH	1.05
Myclobutanil	Fungicide	2.89	Stable in water (pH 4–9) at 25 °C	132
Tebuconazole	Fungicide	3.56	-	36
Bifenthrin	Insecticide	6.6	hydrolysis in alkaline media	0.001
Lambda-cyhalothrin	Insecticide	5.5	hydrolysis in alkaline media	0.9
Beta-cypermethrin	Insecticide	5.8	hydrolysis in strongly alkaline media	0.005
Esfenvalerate	Insecticide	6.24	Rapidly hydrolysis in alkaline media	0.001
Difenoconazole	Fungicide	4.36	-	15
Acetamiprid	Insecticide	0.8	Degrade slowly at pH 9, 45 °C	2950
Imidacloprid	Insecticide	0.57	Stable at pH 5–11	610

* The values of LogP are octanol-water partition coefficient at pH 7, 20 °C.

**Table 2:** GC-MS/MS condition for the identification and quantitation of eight pesticides.

Pesticides	Retention Time(min)	Qualifying Ion Pair	Quantifying Ion Pair	Collision Energy
Chlorpyrifos	9.26	313.09/258	313.09/258	15
		197/169		15
Myclobutanil	11.08	179.06/125.1	179.06/125.1	15
		179.06/152.1		15
Tebuconazole	12.7	250.12/125.1	250.12/125.1	20
		252.13/127.1		20
Bifenthrin	13.29	181.1/166.1	181.1/166.1	15
		181.1/141		22
Lambda-cyhalothrin	14.27	181.04/152	181.04/152	23
		208.05/181		10
Beta-cypermethrin	16.11	181.03/152	181.03/152	25
		163.03/127		10
Esfenvalerate	17.18	167.04/125	167.04/125	10
		167.04/139		10
Difenoconazol	17.57	265.03/202	323.04/265	5
		323.04/265		15

**Table 3:** Liquid chromatography-tandem mass spectrometry parameters of two pesticides.

Pesticides	Retention Time (min)	Precursor Ion	Quantization Ion (Collision Energy)	Identification Ion (Collision Energy)	Fragmentor (V)
Imidacloprid	1.34	256.00	175 (15)	208.9 (15)	120
Acetamiprid	1.40	223.10	126.1 (20)	56.2 (12)	80

**Table 4:** Matrix effects (MEs), calibration curve coefficients (*R*^2^), and LOQs (μg/kg) for ten pesticides in kumquat, cucumber, and spinach (*n* = 5).

Pesticides	Kumquat			Cucumber			Spinach		
	ME	*R* ^2^	LOQ	ME	*R* ^2^	LOQ	ME	*R* ^2^	LOQ
Chlorpyrifos	1.5	0.9999	10	1.5	1.0000	10	1.5	0.9999	10
Myclobutanil	1.5	0.9992	10	1.5	0.9999	10	1.7	0.9998	10
Tebuconazole	2.3	0.9999	10	2.1	1.0000	10	2.6	1.0000	10
Bifenthrin	1.4	0.9995	10	1.4	1.0000	10	1.6	1.0000	10
Lambda-cyhalothrin	1.9	0.9995	10	2.2	0.9999	10	3.8	0.9998	10
Beta-cypermethrin	2	0.9999	10	2.2	0.9995	10	4.1	0.9998	10
Esfenvalerate	2.9	0.9994	10	2.2	0.9999	10	4.9	1.0000	10
Difenoconazole	2.5	0.9998	10	2.3	0.9999	10	4.7	0.996	10
Acetamiprid	0.7	0.9998	10	0.7	1.0000	10	0.5	0.9998	10
Imidacloprid	0.7	0.9998	10	0.8	0.9999	10	0.6	0.9999	10

**Table 5:** Average recoveries and relative standard deviations (RSDs) at three spiked levels in kumquat, cucumber, and spinach (*n* = 5).

Pesticides	Average Recovery (%) (RSD (%))								
	Kumquat (μg/kg)			Cucumber (μg/kg)			Spinach (μg/kg)		
	10	100	500	10	100	500	10	100	500
Chlorpyrifos	94 (4)	88 (9)	92 (4)	99 (2)	91 (4)	99 (2)	93 (8)	94 (4)	91 (1)
Myclobutanil	107 (1)	93 (8)	94 (3)	98 (2)	109 (5)	109 (3)	87 (7)	98 (3)	95 (2)
Tebuconazole	97 (1)	85 (6)	89 (2)	97 (2)	96 (6)	101 (2)	84 (6)	91 (2)	89 (1)
Bifenthrin	100 (2)	93 (6)	94 (5)	94 (1)	101 (4)	102 (3)	91 (5)	96 (2)	92 (1)
Lambda-cyhalothrin	94 (5)	89 (5)	91 (4)	95 (3)	107 (4)	105 (4)	85 (5)	83 (2)	84 (4)
Beta-cypermethrin	91 (5)	86 (4)	84 (1)	103 (6)	104 (6)	107 (1)	94 (2)	99 (2)	79 (4)
Esfenvalerate	94 (3)	82 (3)	84 (4)	118 (2)	107 (5)	110 (3)	92 (5)	96 (4)	78 (4)
Difenoconazole	97 (3)	78 (3)	81 (2)	106 (4)	97 (1)	107 (1)	90 (5)	98 (5)	94 (1)
Acetamiprid	86 (6)	100 (1)	97 (2)	90 (6)	100 (2)	104 (1)	98 (10)	97 (5)	86 (3)
Imidacloprid	91 (3)	102 (3)	98 (1)	91 (7)	102 (1)	104 (1)	103 (9)	104 (7)	91 (3)

**Table 6:** Effect of washing treatments for pesticide removal in kumquat (*n* = 3).

Pesticide	Treatment	Treatment Time (min)							
		5		15		20		30	
		Concentration (mg/kg)	Removal (%)	Concentration (mg/kg)	Removal (%)	Concentration (mg/kg)	Removal (%)	Concentration (mg/kg)	Removal (%)
Chlorpyrifos	Initial deposit	0.48 ± 0.043							
	Tap water	0.45 ± 0.004	7	0.36 ± 0.015	24	0.35 ± 0.005	28	0.41 ± 0.007	14
	2% NaHCO_3_	0.46 ± 0.006	4	0.44 ± 0.010	9	0.43 ± 0.006	11	0.39 ± 0.013	18
	AlEW (pH 10.50)	0.42 ± 0.007	12	0.38 ± 0.0035	21	0.40 ± 0.008	17	0.45 ± 0.012	6
	AlEW (pH 12.35)	0.31 ± 0.035	35	0.33 ± 0.012	31	0.33 ± 0.013	31	0.34 ± 0.007	29
	Ozone solution (0.4 mg/L)	0.43 ± 0.022	10	0.43 ± 0.008	10	0.40 ± 0.010	16	0.36 ± 0.022	24
	Micron calcium solution	0.39 ± 0.021	18	0.30 ± 0.028	37	0.29 ± 0.025	40	0.24 ± 0.038	51
	2% Active oxygen solution	0.40 ± 0.014	17	0.32 ± 0.025	33	0.37 ± 0.007	22	0.33 ± 0.018	32
Myclobutanil	Initial deposit	1.37 ± 0.023							
	Tap water	0.97 ± 0.016	29	0.93 ± 0.019	32	0.92 ± 0.051	33	0.90 ± 0.011	34
	2% NaHCO_3_	1.14 ± 0.036	17	0.89 ± 0.029	35	0.77 ± 0.082	44	0.77 ± 0.029	44
	AlEW (pH 10.50)	1.07 ± 0.040	22	0.81 ± 0.062	41	0.89 ± 0.049	35	1.12 ± 0.034	18
	AlEW (pH 12.35)	0.59 ± 0.077	57	0.58 ± 0.066	58	0.56 ± 0.053	59	0.70 ± 0.063	49
	Ozone solution (0.4 mg/L)	0.84 ± 0.051	39	0.97 ± 0.061	29	0.82 ± 0.019	40	0.69 ± 0.034	50
	Micron calcium solution	1.00 ± 0.060	27	0.64 ± 0.062	53	0.64 ± 0.084	53	0.49 ± 0.054	64
	2% Active oxygen solution	0.88 ± 0.065	36	0.53 ± 0.061	61	0.29 ± 0.071	79	0.48 ± 0.032	65
Tebuconazole	Initial deposit	1.01 ± 0.068							
	Tap water	0.71 ± 0.046	30	0.75 ± 0.007	26	0.70 ± 0.017	31	0.69 ± 0.020	32
	2% NaHCO_3_	0.89 ± 0.020	12	0.53 ± 0.040	48	0.45 ± 0.029	55	0.43 ± 0.030	57
	AlEW (pH 10.50)	0.76 ± 0.041	25	0.58 ± 0.019	43	0.63 ± 0.031	38	0.84 ± 0.006	17
	AlEW (pH 12.35)	0.44 ± 0.055	56	0.42 ± 0.048	58	0.41 ± 0.042	59	0.52 ± 0.051	49
	Ozone solution (0.4 mg/L)	0.54 ± 0.062	47	0.70 ± 0.040	31	0.53 ± 0.031	48	0.37 ± 0.042	63
	Micron calcium solution	0.73 ± 0.036	28	0.48 ± 0.055	52	0.46 ± 0.062	54	0.37 ± 0.072	63
	2% Active oxygen solution	0.66 ± 0.070	35	0.21 ± 0.062	79	0.66 ± 0.041	35	0.31 ± 0.041	69
Bifenthrin	Initial deposit	0.43 ± 0.064							
	Tap water	0.35 ± 0.002	18	0.34 ± 0.013	21	0.32 ± 0.016	26	0.31 ± 0.015	27
	2% NaHCO_3_	0.30 ± 0.007	31	0.24 ± 0.020	45	0.22 ± 0.026	49	0.22 ± 0.006	49
	AlEW (pH 10.50)	0.31 ± 0.009	27	0.34 ± 0.017	22	0.27 ± 0.019	38	0.34 ± 0.005	20
	AlEW (pH 12.35)	0.23 ± 0.032	46	0.23 ± 0.020	46	0.23 ± 0.017	46	0.25 ± 0.011	43
	Ozone solution (0.4 mg/L)	0.26 ± 0.016	40	0.28 ± 0.002	36	0.29 ± 0.036	33	0.22 ± 0.019	50
	Micron calcium solution	0.32 ± 0.009	25	0.25 ± 0.015	42	0.22 ± 0.043	48	0.18 ± 0.020	59
	2% Active oxygen solution	0.25 ± 0.026	43	0.15 ± 0.028	65	0.23 ± 0.009	46	0.15 ± 0.017	64
Lambda-cyhalothrin	Initial deposit	0.48 ± 0.020							
	Tap water	0.38 ± 0.016	21	0.37 ± 0.007	22	0.34 ± 0.013	30	0.34 ± 0.009	30
	2% NaHCO_3_	0.25 ± 0.014	48	0.22 ± 0.026	55	0.20 ± 0.028	59	0.22 ± 0.002	55
	AlEW (pH 10.50)	0.36 ± 0.025	26	0.29 ± 0.025	39	0.36 ± 0.027	25	0.38 ± 0.003	20
	AlEW (pH 12.35)	0.24 ± 0.019	50	0.25 ± 0.045	48	0.20 ± 0.027	59	0.24 ± 0.062	49
	Ozone solution (0.4 mg/L)	0.26 ± 0.023	46	0.24 ± 0.017	50	0.30 ± 0.028	38	0.19 ± 0.029	61
	Micron calcium solution	0.33 ± 0.014	31	0.24 ± 0.016	50	0.22 ± 0.048	54	0.16 ± 0.027	67
	2% Active oxygen solution	0.25 ± 0.032	48	0.12 ± 0.025	74	0.25 ± 0.003	48	0.12 ± 0.016	74
Beta-cypermethrin	Initial deposit	0.23 ± 0.051							
	Tap water	0.16 ± 0.005	30	0.19 ± 0.002	19	0.15 ± 0.008	35	0.15 ± 0.005	34
	2% NaHCO_3_	0.17 ± 0.016	28	0.11 ± 0.010	52	0.12 ± 0.012	47	0.11 ± 0.001	51
	AlEW (pH 10.50)	0.16 ± 0.006	30	0.15 ± 0.004	33	0.15 ± 0.010	33	0.18 ± 0.021	21
	AlEW (pH 12.35)	0.11 ± 0.017	54	0.12 ± 0.028	47	0.10 ± 0.012	56	0.12 ± 0.015	46
	Ozone solution (0.4 mg/L)	0.17 ± 0.003	28	0.14 ± 0.005	37	0.18 ± 0.015	22	0.12 ± 0.009	49
	Micron calcium solution	0.14 ± 0.005	41	0.10 ± 0.009	57	0.09 ± 0.020	61	0.07 ± 0.015	71
	2% Active oxygen solution	0.16 ± 0.018	31	0.08 ± 0.014	67	0.15 ± 0.004	34	0.07 ± 0.011	68
Esfenvalerate	Initial deposit	3.02 ± 0.076							
	Tap water	2.45 ± 0.018	19	2.39 ± 0.070	21	2.36 ± 0.077	22	2.33 ± 0.052	23
	2% NaHCO_3_	2.39 ± 0.039	21	2.05 ± 0.049	32	1.81 ± 0.044	40	1.75 ± 0.066	42
	AlEW (pH 10.50)	2.39 ± 0.012	21	2.30 ± 0.077	24	1.90 ± 0.069	37	2.48 ± 0.080	18
	AlEW (pH 12.35)	1.72 ± 0.009	43	1.45 ± 0.007	52	1.51 ± 0.010	50	1.84 ± 0.008	39
	Ozone solution (0.4 mg/L)	1.99 ± 0.048	34	2.23 ± 0.026	26	1.96 ± 0.012	35	1.69 ± 0.002	44
	Micron calcium solution	2.27 ± 0.073	25	1.93 ± 0.164	36	1.66 ± 0.072	45	1.39 ± 0.038	54
	2% Active oxygen solution	1.99 ± 0.064	34	1.42 ± 0.073	53	1.93 ± 0.014	36	1.45 ± 0.024	52
Difenoconazole	Initial deposit	1.13 ± 0.063							
	Tap water	0.74 ± 0.007	34	0.78 ± 0.042	31	0.70 ± 0.027	38	0.74 ± 0.034	34
	2% NaHCO_3_	0.95 ± 0.057	16	0.65 ± 0.049	42	0.63 ± 0.071	44	0.59 ± 0.039	48
	AlEW (pH 10.50)	0.82 ± 0.030	27	0.68 ± 0.054	40	0.83 ± 0.026	26	0.88 ± 0.015	22
	AlEW (pH 12.35)	0.44 ± 0.065	61	0.47 ± 0.043	58	0.45 ± 0.049	60	0.62 ± 0.012	45
	Ozone solution (0.4 mg/L)	0.74 ± 0.059	34	0.83 ± 0.056	26	0.73 ± 0.069	35	0.63 ± 0.070	44
	Micron calcium solution	0.71 ± 0.028	37	0.48 ± 0.064	57	0.53 ± 0.064	53	0.41 ± 0.041	64
	2% Active oxygen solution	0.70 ± 0.056	38	0.41 ± 0.054	64	0.64 ± 0.061	43	0.47 ± 0.030	58
Acetamiprid	Initial deposit	0.33 ± 0.058							
	Tap water	0.24 ± 0.013	26	0.25 ± 0.013	22	0.21 ± 0.011	34	0.26 ± 0.006	21
	2% NaHCO_3_	0.26 ± 0.020	21	0.23 ± 0.011	30	0.19 ± 0.002	42	0.23 ± 0.015	28
	AlEW (pH 10.50)	0.30 ± 0.006	9	0.20 ± 0.005	37	0.20 ± 0.012	37	0.26 ± 0.021	21
	AlEW (pH 12.35)	0.18 ± 0.022	46	0.23 ± 0.015	30	0.17 ± 0.011	49	0.20 ± 0.021	38
	Ozone solution (0.4 mg/L)	0.25 ± 0.031	22	0.21 ± 0.013	36	0.26 ± 0.020	19	0.19 ± 0.009	42
	Micron calcium solution	0.21 ± 0.008	36	0.18 ± 0.012	46	0.16 ± 0.003	51	0.14 ± 0.011	56
	2% Active oxygen solution	0.23 ± 0.017	30	0.13 ± 0.016	59	0.23 ± 0.027	30	0.15 ± 0.016	53
Imidacloprid	Initial deposit	0.28 ± 0.032							
	Tap water	0.18 ± 0.012	33	0.20 ± 0.011	27	0.17 ± 0.006	39	0.20 ± 0.014	27
	2% NaHCO_3_	0.18 ± 0.016	35	0.16 ± 0.012	42	0.13 ± 0.002	52	0.17 ± 0.008	38
	AlEW (pH 10.50)	0.23 ± 0.010	16	0.18 ± 0.011	35	0.16 ± 0.008	42	0.19 ± 0.000	32
	AlEW (pH 12.35)	0.14 ± 0.019	51	0.18 ± 0.014	35	0.13 ± 0.009	54	0.15 ± 0.020	44
	Ozone solution (0.4 mg/L)	0.18 ± 0.027	33	0.15 ± 0.009	45	0.19 ± 0.018	30	0.18 ± 0.008	33
	Micron calcium solution	0.16 ± 0.008	43	0.13 ± 0.011	52	0.12 ± 0.002	57	0.10 ± 0.008	62
	2% Active oxygen solution	0.17 ± 0.016	40	0.09 ± 0.010	67	0.17 ± 0.019	40	0.10 ± 0.015	62

**Table 7:** Effect of washing treatments for pesticide removal in cucumber (*n* = 3).

Pesticide	Treatment	Treatment Time (min)							
		5		15		20		30	
		Concentration (mg/kg)	Removal (%)	Concentration (mg/kg)	Removal (%)	Concentration (mg/kg)	Removal (%)	Concentration (mg/kg)	Removal (%)
Chlorpyrifos	Initial deposit	0.99 ± 0.034							
	Tap water	0.75 ± 0.040	24	0.83 ± 0.017	16	0.78 ± 0.015	21	0.92 ± 0.017	7
	2% NaHCO_3_	0.82 ± 0.042	17	0.62 ± 0.033	37	0.53 ± 0.035	46	0.56 ± 0.042	43
	AlEW (pH 10.50)	0.79 ± 0.025	20	0.74 ± 0.058	25	0.75 ± 0.038	24	0.75 ± 0.036	24
	AlEW (pH 12.35)	0.87 ± 0.006	12	0.82 ± 0.025	17	0.63 ± 0.021	36	0.72 ± 0.025	27
	Ozone solution	0.75 ± 0.006	24	0.83 ± 0.017	16	0.78 ± 0.045	21	0.82 ± 0.041	17
	Micron calcium solution	0.50 ± 0.034	49	0.64 ± 0.033	35	0.50 ± 0.031	50	0.49 ± 0.021	51
	2% Active oxygen solution	0.82 ± 0.010	17	0.61 ± 0.048	38	0.50 ± 0.064	49	0.58 ± 0.031	41
Myclobutanil	Initial deposit	2.58 ± 0.089							
	Tap water	2.27 ± 0.052	12	2.27 ± 0.065	12	1.91 ± 0.039	26	1.78 ± 0.016	31
	2% NaHCO_3_	2.30 ± 0.086	11	1.78 ± 0.081	31	1.63 ± 0.056	37	2.06 ± 0.071	20
	AlEW (pH 10.50)	2.32 ± 0.047	10	2.27 ± 0.013	12	2.22 ± 0.009	14	2.30 ± 0.055	11
	AlEW (pH 12.35)	2.22 ± 0.052	14	2.14 ± 0.039	17	1.91 ± 0.065	26	2.01 ± 0.081	22
	Ozone solution	2.27 ± 0.059	12	2.27 ± 0.004	12	1.91 ± 0.067	26	1.78 ± 0.068	31
	Micron calcium solution	1.70 ± 0.038	34	2.27 ± 0.078	12	1.50 ± 0.026	42	1.70 ± 0.009	34
	2% Active oxygen solution	2.24 ± 0.054	13	1.78 ± 0.052	31	1.52 ± 0.046	41	1.81 ± 0.024	30
Tebuconazole	Initial deposit	2.22 ± 0.86							
	Tap water	1.98 ± 0.059	11	1.95 ± 0.063	12	1.64 ± 0.069	26	1.58 ± 0.031	29
	2% NaHCO_3_	1.95 ± 0.043	12	1.53 ± 0.068	31	1.35 ± 0.057	39	1.69 ± 0.090	24
	AlEW (pH 10.50)	1.89 ± 0.029	15	1.91 ± 0.059	14	1.84 ± 0.050	17	1.93 ± 0.056	13
	AlEW (pH 12.35)	1.95 ± 0.010	12	1.98 ± 0.063	11	1.60 ± 0.074	28	1.60 ± 0.069	28
	Ozone solution	1.98 ± 0.062	11	1.95 ± 0.068	12	1.64 ± 0.012	26	1.58 ± 0.019	29
	Micron calcium solution	1.44 ± 0.071	35	1.84 ± 0.049	17	1.18 ± 0.046	47	1.40 ± 0.038	37
	2% Active oxygen solution	1.95 ± 0.045	12	1.53 ± 0.070	31	1.33 ± 0.061	40	1.55 ± 0.013	30
Bifenthrin	Initial deposit	0.58 ± 0.044							
	Tap water	0.49 ± 0.059	15	0.48 ± 0.012	18	0.45 ± 0.018	22	0.47 ± 0.035	19
	2% NaHCO_3_	0.46 ± 0.023	21	0.28 ± 0.003	51	0.30 ± 0.032	49	0.45 ± 0.025	22
	AlEW (pH 10.50)	0.31 ± 0.009	47	0.32 ± 0.023	45	0.26 ± 0.021	56	0.27 ± 0.016	53
	AlEW (pH 12.35)	0.24 ± 0.009	58	0.30 ± 0.016	48	0.20 ± 0.016	66	0.22 ± 0.023	62
	Ozone solution	0.41 ± 0.027	30	0.48 ± 0.025	18	0.30 ± 0.011	49	0.48 ± 0.030	17
	Micron calcium solution	0.21 ± 0.020	64	0.40 ± 0.006	31	0.19 ± 0.030	67	0.33 ± 0.010	43
	2% Active oxygen solution	0.45 ± 0.009	22	0.34 ± 0.026	42	0.25 ± 0.012	57	0.40 ± 0.030	31
Lambda-Cyhalothrin	Initial deposit	0.64 ± 0.013							
	Tap water	0.56 ± 0.058	13	0.55 ± 0.007	14	0.53 ± 0.006	17	0.51 ± 0.030	21
	2% NaHCO_3_	0.33 ± 0.042	48	0.30 ± 0.003	53	0.30 ± 0.032	53	0.33 ± 0.036	48
	AlEW (pH 10.50)	0.44 ± 0.011	32	0.41 ± 0.037	36	0.32 ± 0.023	50	0.36 ± 0.023	43
	AlEW (pH 12.35)	0.41 ± 0.027	36	0.52 ± 0.035	19	0.31 ± 0.005	51	0.36 ± 0.015	43
	Ozone solution	0.37 ± 0.021	42	0.44 ± 0.025	31	0.31 ± 0.010	52	0.51 ± 0.031	21
	Micron calcium solution	0.15 ± 0.018	77	0.20 ± 0.014	68	0.11 ± 0.022	83	0.13 ± 0.011	79
	2% Active oxygen solution	0.5 ± 0.010	13	0.37 ± 0.025	42	0.27 ± 0.009	58	0.47 ± 0.037	26
Beta-cypermethrin	Initial deposit	0.59 ± 0.025							
	Tap water	0.54 ± 0.029	9	0.48 ± 0.016	19	0.45 ± 0.031	24	0.48 ± 0.032	18
	2% NaHCO_3_	0.55 ± 0.012	7	0.35 ± 0.023	40	0.28 ± 0.008	53	0.39 ± 0.021	34
	AlEW (pH 10.50)	0.39 ± 0.025	34	0.35 ± 0.030	40	0.31 ± 0.017	48	0.31 ± 0.008	48
	AlEW (pH 12.35)	0.38 ± 0.020	36	0.41 ± 0.009	31	0.27 ± 0.015	54	0.30 ± 0.007	49
	Ozone solution	0.35 ± 0.003	40	0.40 ± 0.029	33	0.32 ± 0.011	46	0.46 ± 0.016	22
	Micron calcium solution	0.12 ± 0.017	80	0.15 ± 0.019	74	0.09 ± 0.017	85	0.09 ± 0.013	85
	2% Active oxygen solution	0.48 ± 0.041	19	0.30 ± 0.005	49	0.29 ± 0.033	51	0.26 ± 0.026	56
Esfenvalerate	Initial deposit	2.58 ± 0.071							
	Tap water	2.14 ± 0.068	17	2.09 ± 0.063	19	2.12 ± 0.041	18	2.17 ± 0.065	16
	2% NaHCO_3_	1.86 ± 0.039	28	1.08 ± 0.025	58	1.21 ± 0.073	53	2.24 ± 0.043	13
	AlEW (pH 10.50)	1.44 ± 0.024	44	1.44 ± 0.079	44	1.21 ± 0.014	53	1.29 ± 0.032	50
	AlEW (pH 12.35)	1.11 ± 0.048	57	1.32 ± 0.026	49	0.77 ± 0.064	70	0.98 ± 0.062	62
	Ozone solution	2.14 ± 0.068	17	1.96 ± 0.021	24	1.60 ± 0.062	38	1.57 ± 0.069	39
	Micron calcium solution	0.41 ± 0.060	84	1.26 ± 0.016	51	0.36 ± 0.078	86	0.70 ± 0.039	73
	2% Active oxygen solution	2.09 ± 0.015	19	1.29 ± 0.076	50	0.95 ± 0.028	63	1.63 ± 0.002	37
Difenoconazole	Initial deposit	1.35 ± 0.064							
	Tap water	1.03 ± 0.035	24	1.13 ± 0.043	16	0.90 ± 0.034	33	1.00 ± 0.010	26
	2% NaHCO_3_	1.08 ± 0.073	20	0.76 ± 0.034	44	0.68 ± 0.045	50	0.86 ± 0.078	36
	AlEW (pH 10.50)	1.00 ± 0.030	26	1.08 ± 0.039	20	0.80 ± 0.049	41	0.93 ± 0.051	31
	AlEW (pH 12.35)	0.97 ± 0.036	28	0.90 ± 0.000	33	0.88 ± 0.059	35	0.80 ± 0.054	41
	Ozone solution	1.03 ± 0.009	24	1.13 ± 0.016	16	0.90 ± 0.058	33	1.00 ± 0.019	26
	Micron calcium solution	0.50 ± 0.060	63	0.70 ± 0.026	48	0.45 ± 0.030	67	0.47 ± 0.019	65
	2% Active oxygen solution	1.09 ± 0.068	19	0.74 ± 0.050	45	0.63 ± 0.047	53	0.74 ± 0.045	45
Acetamiprid	Initial deposit	1.65 ± 0.053							
	Tap water	1.45 ± 0.071	12	1.37 ± 0.072	17	1.16 ± 0.065	30	1.34 ± 0.051	19
	2% NaHCO_3_	1.24 ± 0.007	25	1.12 ± 0.061	32	0.99 ± 0.017	40	1.30 ± 0.070	21
	AlEW (pH 10.50)	1.52 ± 0.008	8	1.40 ± 0.037	15	1.40 ± 0.010	15	1.47 ± 0.031	11
	AlEW (pH 12.35)	1.50 ± 0.032	9	1.37 ± 0.013	17	1.42 ± 0.036	14	1.45 ± 0.036	12
	Ozone solution	1.45 ± 0.017	12	1.37 ± 0.021	17	1.16 ± 0.031	30	1.34 ± 0.007	19
	Micron calcium solution	1.19 ± 0.020	28	1.44 ± 0.076	13	1.04 ± 0.014	37	1.22 ± 0.027	26
	2% Active oxygen solution	1.30 ± 0.061	21	0.94 ± 0.044	43	0.84 ± 0.003	49	0.87 ± 0.004	47
Imidacloprid	Initial deposit	1.82 ± 0.061							
	Tap water	1.66 ± 0.062	9	1.57 ± 0.055	14	1.35 ± 0.048	26	1.49 ± 0.009	18
	2% NaHCO_3_	1.35 ± 0.063	26	1.18 ± 0.044	35	1.07 ± 0.021	41	1.46 ± 0.050	20
	AlEW (pH 10.50)	1.66 ± 0.010	9	1.51 ± 0.016	17	1.57 ± 0.009	14	1.60 ± 0.018	12
	AlEW (pH 12.35)	1.40 ± 0.059	23	1.47 ± 0.022	19	1.20 ± 0.036	34	1.55 ± 0.007	15
	Ozone solution	1.66 ± 0.031	9	1.57 ± 0.029	14	1.35 ± 0.038	26	1.49 ± 0.009	18
	Micron calcium solution	1.38 ± 0.024	24	1.55 ± 0.077	15	1.18 ± 0.037	35	1.33 ± 0.050	27
	2% Active oxygen solution	1.49 ± 0.063	18	1.00 ± 0.036	45	0.91 ± 0.009	50	0.95 ± 0.016	48

**Table 8:** Effect of washing treatments for pesticide removal in spinach (*n* = 3).

Pesticide	Treatment	Treatment Time (min)							
		5		15		20		30	
		Average concentration (mg/kg)	Removal (%)	Average concentration (mg/kg)	Removal (%)	Average concentration (mg/kg)	Removal (%)	Average concentration(mg/kg)	Removal (%)
Chlorpyrifos	Initial deposit	1.13 ± 0.047							
	Tap water	1.08 ± 0.023	4	1.04 ± 0.046	8	1.03 ± 0.032	9	0.94 ± 0.006	17
	2% NaHCO_3_	0.92 ± 0.064	19	0.71 ± 0.011	37	0.78 ± 0.043	31	0.86 ± 0.049	24
	AlEW (pH 10.50)	0.92 ± 0.019	19	0.85 ± 0.040	25	0.80 ± 0.035	29	0.79 ± 0.044	30
	AlEW (pH 12.35)	0.94 ± 0.046	17	0.78 ± 0.025	31	0.84 ± 0.068	26	0.89 ± 0.067	21
	Ozone solution	0.94 ± 0.041	17	0.57 ± 0.057	50	0.73 ± 0.041	35	0.53 ± 0.038	53
	Micron calcium	0.92 ± 0.039	19	0.76 ± 0.069	33	0.76 ± 0.007	33	0.67 ± 0.005	41
	2% Active oxygen	0.54 ± 0.064	52	0.60 ± 0.095	47	0.72 ± 0.083	36	0.58 ± 0.072	49
Myclobutanil	Initial deposit	2.12 ± 0.052							
	Tap water	1.82 ± 0.082	14	1.55 ± 0.044	27	1.51 ± 0.056	29	1.23 ± 0.026	42
	2% NaHCO_3_	1.63 ± 0.060	23	1.06 ± 0.019	50	1.27 ± 0.045	40	1.44 ± 0.035	32
	AlEW (pH 10.50)	1.51 ± 0.019	29	1.51 ± 0.031	29	1.42 ± 0.039	33	1.59 ± 0.014	25
	AlEW (pH 12.35)	1.74 ± 0.059	18	1.27 ± 0.017	40	1.29 ± 0.027	39	1.46 ± 0.062	31
	Ozone solution	1.78 ± 0.007	16	0.76 ± 0.080	64	1.06 ± 0.075	50	0.59 ± 0.050	72
	Micron calcium	1.40 ± 0.031	34	1.06 ± 0.029	50	1.08 ± 0.014	49	0.89 ± 0.006	58
	2% Active oxygen	0.78 ± 0.018	63	0.81 ± 0.014	62	1.31 ± 0.026	38	0.91 ± 0.011	57
Tebuconazole	Initial deposit	1.61 ± 0.078							
	Tap water	1.38 ± 0.063	14	1.18 ± 0.020	27	1.16 ± 0.045	28	0.95 ± 0.037	41
	2% NaHCO_3_	1.21 ± 0.039	25	0.82 ± 0.022	49	0.93 ± 0.063	42	1.08 ± 0.055	33
	AlEW (pH 10.50)	1.09 ± 0.043	32	1.08 ± 0.054	33	1.05 ± 0.041	35	1.14 ± 0.027	29
	AlEW (pH 12.35)	1.30 ± 0.017	19	0.97 ± 0.008	40	0.97 ± 0.072	40	1.08 ± 0.015	33
	Ozone solution	1.21 ± 0.039	25	0.56 ± 0.057	65	0.79 ± 0.038	51	0.43 ± 0.033	73
	Micron calcium	1.05 ± 0.032	35	0.77 ± 0.056	52	0.79 ± 0.004	51	0.66 ± 0.023	59
	2% Active oxygen	0.56 ± 0.065	65	0.61 ± 0.087	62	1.00 ± 0.025	38	0.64 ± 0.010	60
Bifenthrin	Initial deposit	1.12 ± 0.061							
	Tap water	1.08 ± 0.006	4	0.81 ± 0.032	28	0.82 ± 0.018	27	0.73 ± 0.011	35
	2% NaHCO_3_	0.92 ± 0.034	18	0.68 ± 0.007	39	0.83 ± 0.054	26	0.88 ± 0.031	21
	AlEW (pH 10.50)	0.77 ± 0.030	31	0.78 ± 0.079	30	0.82 ± 0.063	27	0.86 ± 0.032	23
	AlEW (pH 12.35)	0.80 ± 0.008	29	0.72 ± 0.025	36	0.75 ± 0.040	33	0.87 ± 0.071	22
	Ozone solution	0.96 ± 0.040	14	0.47 ± 0.032	58	0.74 ± 0.031	34	0.43 ± 0.017	62
	Micron calcium	0.84 ± 0.039	25	0.68 ± 0.029	39	0.64 ± 0.014	43	0.58 ± 0.018	48
	2% Active oxygen	0.50 ± 0.053	55	0.47 ± 0.070	58	0.73 ± 0.076	35	0.48 ± 0.052	57
Lambda-Cyhalothrin	Initial deposit	0.86 ± 0.040							
	Tap water	0.82 ± 0.005	5	0.73 ± 0.039	15	0.76 ± 0.042	12	0.76 ± 0.054	12
	2% NaHCO_3_	0.46 ± 0.052	47	0.29 ± 0.014	66	0.34 ± 0.028	61	0.36 ± 0.014	58
	AlEW (pH 10.50)	0.62 ± 0.007	28	0.58 ± 0.043	33	0.44 ± 0.055	49	0.49 ± 0.038	43
	AlEW (pH 12.35)	0.52 ± 0.043	39	0.48 ± 0.022	44	0.45 ± 0.032	48	0.49 ± 0.062	43
	Ozone solution	0.41 ± 0.041	52	0.28 ± 0.019	68	0.39 ± 0.029	55	0.28 ± 0.009	67
	Micron calcium	0.47 ± 0.067	45	0.45 ± 0.071	48	0.48 ± 0.011	44	0.49 ± 0.043	43
	2% Active oxygen	0.26 ± 0.046	70	0.27 ± 0.018	69	0.48 ± 0.033	44	0.25 ± 0.045	71
Beta-cypermethrin	Initial deposit	0.66 ± 0.013							
	Tap water	0.61 ± 0.017	7	0.54 ± 0.023	18	0.57 ± 0.039	13	0.61 ± 0.009	8
	2% NaHCO_3_	0.32 ± 0.051	51	0.22 ± 0.021	66	0.24 ± 0.021	63	0.27 ± 0.24	59
	AlEW (pH 10.50)	0.55 ± 0.037	17	0.48 ± 0.023	27	0.34 ± 0.026	48	0.41 ± 0.018	38
	AlEW (pH 12.35)	0.35 ± 0.064	47	0.32 ± 0.025	52	0.28 ± 0.028	57	0.33 ± 0.053	50
	Ozone solution	0.36 ± 0.032	45	0.22 ± 0.038	67	0.29 ± 0.076	56	0.23 ± 0.017	65
	Micron calcium	0.26 ± 0.053	61	0.26 ± 0.059	61	0.29 ± 0.008	56	0.31 ± 0.040	53
	2% Active oxygen	0.19 ± 0.038	71	0.20 ± 0.031	69	0.34 ± 0.027	48	0.19 ± 0.045	71
Esfenvalerate	Initial deposit	2.11 ± 0.046							
	Tap water	1.54 ± 0.068	27	1.52 ± 0.010	28	1.52 ± 0.028	28	1.90 ± 0.057	10
	2% NaHCO_3_	1.01 ± 0.075	52	0.55 ± 0.046	74	0.65 ± 0.053	69	0.72 ± 0.069	66
	AlEW (pH 10.50)	1.48 ± 0.008	30	1.37 ± 0.006	35	0.87 ± 0.042	59	1.10 ± 0.045	48
	AlEW (pH 12.35)	1.12 ± 0.016	47	1.01 ± 0.068	52	0.91 ± 0.021	57	1.06 ± 0.014	50
	Ozone solution	1.10 ± 0.083	48	0.42 ± 0.082	80	0.74 ± 0.071	65	0.46 ± 0.047	78
	Micron calcium	0.89 ± 0.15	58	0.91 ± 0.002	57	0.89 ± 0.062	58	0.97 ± 0.013	54
	2% Active oxygen	0.40 ± 0.011	81	0.42 ± 0.055	80	0.99 ± 0.079	53	0.42 ± 0.036	80
Difenoconazole	Initial deposit	1.64 ± 0.039							
	Tap water	1.43 ± 0.033	13	1.30 ± 0.054	21	1.28 ± 0.043	22	1.08 ± 0.050	34
	2% NaHCO_3_	1.23 ± 0.057	25	0.89 ± 0.032	46	1.02 ± 0.074	38	1.18 ± 0.064	28
	AlEW (pH 10.50)	1.16 ± 0.043	29	1.10 ± 0.015	33	1.03 ± 0.036	37	1.28 ± 0.042	22
	AlEW (pH 12.35)	1.34 ± 0.058	18	1.03 ± 0.020	37	1.08 ± 0.032	34	1.20 ± 0.017	27
	Ozone solution	1.28 ± 0.040	22	0.62 ± 0.011	62	0.90 ± 0.037	45	0.52 ± 0.033	68
	Micron calcium	0.61 ± 0.074	63	0.89 ± 0.077	46	0.85 ± 0.012	48	0.74 ± 0.007	55
	2% Active oxygen	0.62 ± 0.071	62	0.64 ± 0.004	61	1.00 ± 0.012	39	0.72 ± 0.014	56
Acetamiprid	Initial deposit	1.57 ± 0.072							
	Tap water	1.37 ± 0.028	13	1.07 ± 0.001	32	1.13 ± 0.051	28	0.99 ± 0.059	37
	2% NaHCO_3_	1.18 ± 0.066	25	0.94 ± 0.004	40	1.05 ± 0.054	33	1.11 ± 0.028	29
	AlEW (pH 10.50)	1.16 ± 0.045	26	1.18 ± 0.016	25	1.11 ± 0.028	29	1.30 ± 0.067	17
	AlEW (pH 12.35)	1.33 ± 0.062	15	0.99 ± 0.008	37	0.97 ± 0.026	38	1.04 ± 0.065	34
	Ozone solution	1.38 ± 0.043	12	0.71 ± 0.016	55	0.86 ± 0.061	45	0.57 ± 0.049	64
	Micron calcium	1.24 ± 0.021	21	1.05 ± 0.079	33	0.96 ± 0.031	39	1.04 ± 0.072	34
	2% Active oxygen	0.79 ± 0.081	50	0.80 ± 0.032	49	1.07 ± 0.096	32	0.82 ± 0.056	48
Imidacloprid	Initial deposit	1.82 ± 0.033							
	Tap water	1.89 ± 0.050	9	1.26 ± 0.013	31	1.37 ± 0.024	25	1.20 ± 0.014	34
	2% NaHCO_3_	1.29 ± 0.018	29	0.98 ± 0.008	46	1.13 ± 0.039	38	1.29 ± 0.052	29
	AlEW (pH 10.50)	1.37 ± 0.046	25	1.42 ± 0.017	22	1.27 ± 0.051	30	1.64 ± 0.012	10
	AlEW (pH 12.35)	1.53 ± 0.055	16	1.11 ± 0.007	39	1.15 ± 0.062	37	1.24 ± 0.081	32
	Ozone solution	1.55 ± 0.081	15	0.84 ± 0.049	54	1.02 ± 0.047	44	0.67 ± 0.028	63
	Micron calcium	1.44 ± 0.052	21	1.18 ± 0.064	35	1.09 ± 0.025	40	1.20 ± 0.050	34
	2% Active oxygen	0.95 ± 0.074	48	0.96 ± 0.057	47	1.26 ± 0.004	31	0.95 ± 0.061	48

**Table 9:** The optimal treatments of pesticides in kumquat, cucumber, and spinach.

**The Total Optimal Conditions**	**Kumquat**	**Cucumber**	**Spinach**
	2% Active oxygen (15 min)	Micron calcium (20 min)	Ozone solution (30 min)
